# Development and validation of an artificial intelligence model for the classification of hip fractures using the AO-OTA framework

**DOI:** 10.2340/17453674.2024.40949

**Published:** 2024-06-18

**Authors:** Ehsan AKBARIAN, Mehrgan MOHAMMADI, Emilia TIALA, Oscar LJUNGBERG, Ali SHARIF RAZAVIAN, Martin MAGNÈLI, Max GORDON

**Affiliations:** Department of Clinical Sciences, Karolinska Institutet, Danderyd University Hospital, Stockholm, Sweden

## Abstract

**Background and purpose:**

Artificial intelligence (AI) has the potential to aid in the accurate diagnosis of hip fractures and reduce the workload of clinicians. We primarily aimed to develop and validate a convolutional neural network (CNN) for the automated classification of hip fractures based on the 2018 AO-OTA classification system. The secondary aim was to incorporate the model’s assessment of additional radiographic findings that often accompany such injuries.

**Methods:**

6,361 plain radiographs of the hip taken between 2002 and 2016 at Danderyd University Hospital were used to train the CNN. A separate set of 343 radiographs representing 324 unique patients was used to test the performance of the network. Performance was evaluated using area under the curve (AUC), sensitivity, specificity, and Youden’s index.

**Results:**

The CNN demonstrated high performance in identifying and classifying hip fracture, with AUCs ranging from 0.76 to 0.99 for different fracture categories. The AUC for hip fractures ranged from 0.86 to 0.99, for distal femur fractures from 0.76 to 0.99, and for pelvic fractures from 0.91 to 0.94. For 29 of 39 fracture categories, the AUC was ≥ 0.95.

**Conclusion:**

We found that AI has the potential for accurate and automated classification of hip fractures based on the AO-OTA classification system. Further training and modification of the CNN may enable its use in clinical settings.

Hip fractures are prevalent in the elderly and are associated with increased morbidity and mortality [1,2]. Traditional imaging techniques like radiographs often suffice for diagnosis; however, MRI and CT scan are necessary when radiographs are inconclusive or when a fracture is suspected despite normal radiographic results [3]. Accurate and timely diagnosis of a hip fracture is paramount, especially in settings where experienced radiologists are not readily available to interpret plain radiographs [4]. This is especially crucial for hip fracture classification, as the correct implant type hinges on the diagnosis. In this context, computer-aided diagnosis (CAD) systems have the potential to assist clinicians in making accurate diagnoses.

Machine learning (ML) is an artificial intelligence technology where computers learn from data to spot patterns and solve problems. Deep learning (DL), a specialized branch of ML, uses layers of processing, similar to our brain’s neurons, to analyze complex data. This technology shines in tasks like examining radiographs to identify fractures. Some studies [5,6] have shown that DL, and in particular a type called convolutional neural network (CNN), can improve the accuracy of fracture detection. This advancement could potentially ease the workload of clinicians and reduce the dependence on costly imaging tests, as highlighted in some previous studies [7,8].

Previous work has applied DL to fractures such as those of the ankle, wrist, and knee, with favorable outcomes [9,10]. Recent studies [11-14] have explored various aspects of hip fracture classification, including the application of deep learning techniques; yet a comprehensive approach integrating the complexities of real-world scenarios such as radiological comorbidities, joint arthritis, tumors, and medical implants remains a challenge.

Our study aims to develop a CNN model for the AO-OTA 2018 Classification system [15] that we have extended with extra classes of clinically relevance.

## Methods

### Study design

This cross-sectional validation study evaluates a diagnostic method based on a neural network analyzing retrospectively collected radiographic examinations of the hip joint for both the presence and type of fracture. Radiographic series of the hip taken between 2002 and 2016 were extracted from Danderyd University Hospital’s Picture Archiving and Communication System (PACS) in Stockholm, Sweden. Random subsets of image series were curated, including both images annotated with phrases by radiologists that suggest potential fractures and those without such indications. A weighted selection process was employed to ensure a balanced representation of images with fractures and those without. These images were then uploaded to a cloud-based server, facilitating the use of the AO-OTA 2018 classification system for image analysis on any computer. To enhance the transparency and reproducibility of our research, we adhered to the guidelines set forth by the Transparent Reporting of a multivariable Prediction model for Individual Prognosis Or Diagnosis (TRIPOD) throughout the design, analysis, and reporting of our study [16].

### Inclusion and exclusion criteria

***Inclusion criteria.*** Pelvic and hip radiographs from patients with both trauma and non-trauma protocols were included. Projections were not standardized and there was no patient overlap between the training and test sets.

**Figure 1:**
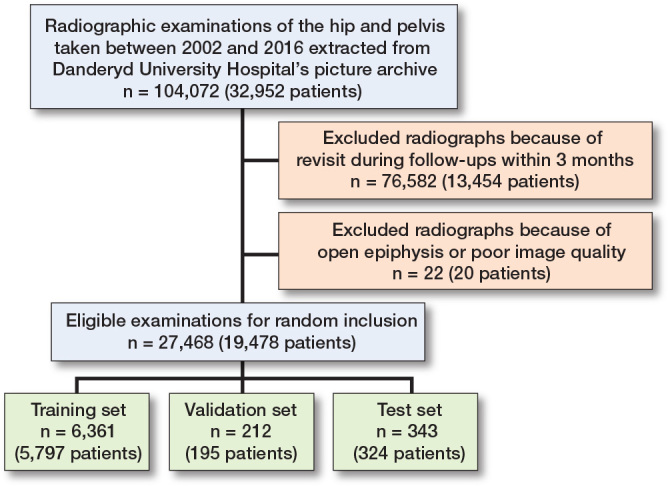
Flow diagram for study inclusion and exclusion.

***Exclusion criteria.*** Radiographs with open epiphyses were rejected. Radiographs taken as follow-up of patients within 3 months were excluded to prevent the network from processing duplicate images (Figure 1).

### Classification method

**Figure 2:**
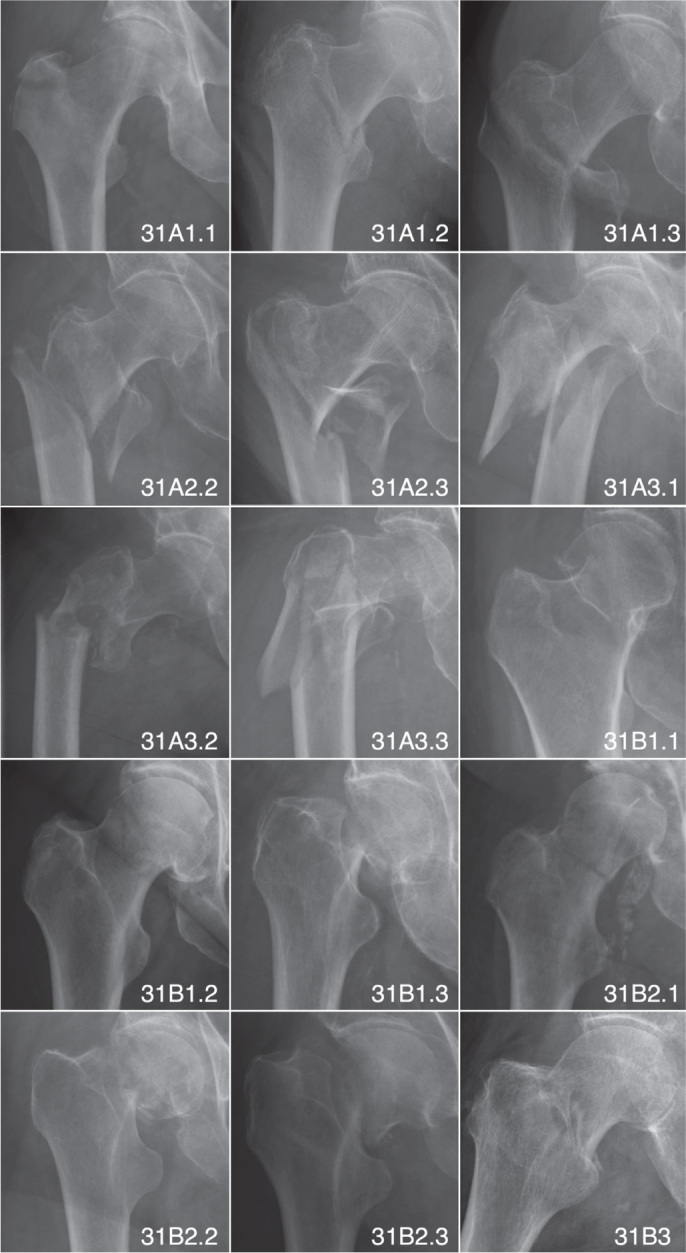
Examples of hip fractures classified by AO-OTA classification system.

The AO-OTA fracture and dislocation classification system categorizes fractures by bone segments and morphology based on a hierarchy of trauma energy and treatment difficulty [15]. Figure 2 shows some of the cases in our dataset classified according to the AO-OTA classification system.

Fracture classifications were strategically merged when treatment modalities converged, simplifying the dataset without compromising clinical relevance. Classes A12 and B3, as well as A22, A23, and A33 fractures, were combined due to their common treatment approach. Displaced B3 fractures were specifically grouped due to their unique treatment considerations. These groupings followed standard clinical protocols, as substantiated by existing literature and guidelines [17-19]. To enhance the clinical applicability of the model, we also incorporated custom modifiers for specific AO classes, including “displaced,” “trochanter major,” and “long minor” subcategories to capture additional clinically relevant details. We defined “displaced” as fractures with more than 30 degrees of angulation and more than 2 cm of shortening, “trochanter major” as fractures with separated trochanter major, and “long minor” as fractures with the trochanter minor segment extending 2 cm or more below the distal limit of the fragment.

### Data sets

***Training set.*** The training dataset, encompassing 6,361 images (5,797 unique patients), was rigorously labeled for the presence of fractures, osteoarthrosis, and other pathologies such as tumors and implants. This initial classification, conducted by medical students under the senior author‘s supervision, was further refined through a review process involving a senior orthopedic surgeon for ambiguous cases. Active learning principles guided the selection of cases for the network‘s calibration, where it was progressively trained on images with both high likelihood and high ambiguity of fractures. Discrepancies between the network’s predictions and the initial labels prompted a thorough re-evaluation, leading to reclassification where necessary to ensure the accuracy of the training dataset, critical for the effectiveness of the subsequent CNN model training.

***Test set.*** A set of 343 exams (324 unique patients) was used as the final test to assess the network’s performance. There was no image overlap between the test and training datasets. 2 experienced senior orthopedic surgeons, blinded to the network’s predictions and initially independent of each other, classified the test set. Images with differing classifications were revisited, and consensus was reached after mutual discussion.

### Neural network setup

A supervised learning method was employed to train a 39-layer ResNet CNN architecture as our network. This architecture used batch normalization for each convolutional layer and adaptive max pool. The training was divided into multiple sessions with different regularizations for overfitting control. The network was trained in 2 phases: regular dropout without any noise, and dropout with added white noise and random block dropout. The learning rate was reset between sessions.

The AI model developed the versatility to identify over 1,000 diagnostic categories, spanning multiple anatomical regions. For the purpose of our focused analysis on hip fractures, the model was trained on 213 hip-specific categories, including 25 that were aggregated to facilitate a more streamlined evaluation process. Our reporting methodology intentionally omits the distinction between left- and right-sided fractures, opting to record a fracture if it was discernible on either side. Moreover, we have incorporated additional pathologies pertinent to hip conditions such as implants, avascular necrosis, and osteoarthritis into our assessment. This targeted reporting strategy was governed by the depth of data available for each category and is comprehensively represented in the study’s tabulated findings.

### Input images

The dataset of images and their labeled outcomes were introduced to the network individually. Each radiograph was automatically cropped to the active image area (removing any black border) and resized to a maximum of 256 pixels. Padding was added to the rectangular image, resulting in a square format of 256 x 256 pixels for input to the network.

### Statistics

Network performance was assessed using the area under the curve (AUC) as the primary outcome measure, with 95% confidence intervals (CIs). Sensitivity, specificity, and Youden’s index were secondary outcome measures. The proportion of correctly detected fractures was estimated using AUC, with frequency-weighted AUC calculated for summarized groups of categories:


$$AUC_{weighted}=\frac{\sum_{i=1}^{categories}AUC_{i}n_{i}}{\sum_{i=1}^{categories}n_{i}}$$

An AUC value of 1 indicates perfect prediction, while an AUC of 0.5 represents no better prediction than random chance. Generally, an AUC of 0.7–0.8 is considered acceptable, 0.8–0.9 is considered good or very good, and ≥ 0.9 is considered outstanding [20]. Youden’s Index (J), defined as J = sensitivity + specificity – 1, is another measure used in conjunction with the receiver operating curve and ranges from 0 to 1.

Given the numerous categories, a weighted mean of each measure was presented, incorporating all subclasses. For example, A-types included not only the A-type but all available subgroups in one measure. Weighting was based on the number of positive cases, minimizing the influence of small categories that may perform well by chance on the weighted mean.

In our study, we quantified the agreement between the 2 senior surgeons on the classification of hip fractures using Cohen’s kappa. This statistical measure was critical, as it highlighted areas where the surgeons’ assessments diverge, necessitating consensus discussions to ensure reliability in the test set evaluations. The network was implemented and trained using PyTorch (v. 1.10; https://pytorch.org/), and statistical analysis was conducted using R (4.0.0; R Foundation for Statistical Computing, Vienna, Austria).

### Ethics, data sharing, funding, and disclosures

The research was approved by the Swedish Ethical Review Authority (dnr: 2014/453-31/3). This project was supported by grants provided by Region Stockholm (ALF project) that have enabled both research time and computational resources for MG and ASR. The funding bodies played no role in the design of the study and collection, analysis, and interpretation of data or in writing the manuscript. Open access funding was provided by the Karolinska Institute. Authors report no conflict of interests. Complete disclosure of interest forms according to ICMJE are available on the article page, doi: 10.2340/17453674.2024.40949

## Results

### Fracture categories

In the test set, there were 188 cases (55%) and in the training set 3,223 cases (49%). Hip fractures were the most common type in both sets, with 156 cases (45%) in the test set and 2,786 cases (42%) in the training set. Roughly half of examined cases in both the test set and training set had a fracture (Table 1).

**Table 1:** Distribution of cases in training and test sets. Values are count (%) within the same category for each set.

	Test set			Training set		
	Yes	Maybe	No	Yes	Maybe	No
All fractures	188 (55)	4 (1.2)	151 (44)	3,223 (49)	89 (1.4)	3,249 (49)
Hip	156 (45)	2 (0.6)	185 (54)	2,786 (42	20 (0.3)	3,742 (57)
A	72 (21)	1 (0.3)	270 (79)	1,365 (21	8 (0.1)	5,188 (79)
.1	34 (9.9)	1 (0.3)	308 (90)	694 (11	8 (0.1)	5,859 (89)
..1	9 (2.6)	1 (0.3)	333 (97)	91 (1.4	8 (0.1)	6,462 (98)
..2	11 (3.2)		332 (97)	326 (5.0		6,235 (95)
…displaced	2 (0.6)		341 (99)	125 (1.9		6,423 (98)
…trochanter major	3 (0.9)		340 (99)	197 (3.0		6,351 (97)
..3	14 (4.1)		329 (96)	277 (4.2		6,284 (96)
…displaced	9 (2.6)		334 (97)	184 (2.8		6,364 (97)
…trochanter major	8 (2.3)		335 (98)	154 (4.0		3,706 (96)
.2	23 (6.7)		320 (93)	414 (6.3		6,147 (94)
..2	16 (4.7)		327 (95)	306 (4.7		6,255 (95)
…displaced	9 (2.6)		334 (97)	220 (3.4		6,328 (97)
…long minor	5 (1.5)		338 (98)	122 (1.9		6,426 (98)
..3	7 (2.0)		336 (98)	108 (1.6		6,453 (98)
.3	15 (4.4)		328 (96)	257 (3.9		6,304 (96)
..1	3 (0.9)		340 (99)	41 (0.6		6,520 (99)
..2	1 (0.3)		342 (100)	26 (0.4		6,535 (100)
..3	11 (3.2)		332 (97)	190 (2.9		6,371 (97)
…diaphysis	4 (1.2)		339 (99)	60 (0.9		6,488 (99)
…trochanter major	8 (2.3)		335 (98)	115 (1.8		6,233 (98)
B	84 (24)	1 (0.3)	258 (75)	1429 (22)	12 (0.2)	5,104 (78)
.1	40 (12)	1 (0.3)	302 (88)	741 (11)	9 (0.1)	5,811 (89)
..1	5 (1.5)		338 (98)	215 (3.3)	2 (0.0)	6,344 (97)
..2	3 (0.9)	1 (0.3)	339 (99)	69 (1.1)	6 (0.1)	6,486 (99)
..3	32 (9.3)		311 (91)	456 (7.0)	1 (0.0)	6,104 (93)
.2	40 (12)		303 (88)	614 (9.4)	3 (0.0)	5,944 (91)
..1	10 (2.9)		333 (97)	207 (3.2)	2 (0.0)	6,352 (97)
…displaced	8 (2.3)		335 (98)	156 (2.4)		64,05 (98)
..2	18 (5.2)		325 (95)	177 (2.7)		6,384 (97)
…displaced	15 (4.4)		328 (96)	169 (2.6)		6,392 (97)
..3	12 (3.5)		331 (96)	229 (3.5)	1 (0.0)	6,331 (96)
…displaced	10 (2.9)		333 (97)	202 (3.1)		6,359 (97)
.3	4 (1.2)		339 (99)	74 (1.1)		6,487 (99)
Femur	14 (4.1)	1 (0.3)	328 (96)	39 (0.6)		6,309 (99)
Acetabulum	1 (0.3)		342 (100)	89 (1.4)	4 (0.1)	6,455 (99)
Pelvis	18 (5.2)	1 (0.3)	324 (94)	291 (4.4)	15 (0.2)	6,242 (95)

Most common anatomical distributions of fractures based on the AO-OTA classification. The letter corresponds to fracture type, first number to group, second number to subgroup.

The most common AO-OTA fracture type was the trochanteric region fracture. In this category, the A1 and the A2 groups and subgroups were distributed evenly in both training and test sets. However, both were almost 3 times larger than the A3 groups in the training set and twice the size of the A3 group in the test set. The patient distribution observed in our study aligns with established patterns documented within the wider hip fracture literature [18].

Inter-rater reliability analysis using Cohen’s kappa demonstrated a high degree of agreement between the senior orthopedic surgeons for the classification of hip fractures. The kappa value for identifying fractures was 0.91 (0.87–0.96), indicating excellent agreement. For base hip fractures and category A fractures, kappa values were 0.93, CI 0.89–0.97, and 0.88, CI 0.82–0.95, respectively, reflecting almost perfect agreement. Disparities arose in specific subcategories, with moderate agreement observed (kappa: 0.59 for A1; CI 0.44–0.73). Particularly challenging were subcategories such as trochanter major classifications and certain displaced fractures (some subgroups of A2 and A3), where kappa values occasionally dropped below 0.30, necessitating case-by-case consensus to resolve discrepancies. A detailed table (Table 2, see Appendix) providing a comprehensive breakdown of the interobserver Cohen’s kappa values across all fracture subcategories is included in the Appendix accompanying this article.

**Table 2:** Inter-observer agreement between the 2 orthopedic surgeons

	Cohen’s kappa (CI)
General fracture	0.91 (0.87 to 0.96)
Hip	
Base	0.93 (0.89 to 0.97)
A	0.88 (0.82 to 0.95)
.1	0.59 (0.44 to 0.73)
..1	0.56 (0.25 to 0.88)
..2	0.38 (0.13 to 0.63)
.. → displaced	0.28 (–0.16 to 0.72)
.. → trochanter major	0.14 (–0.13 to 0.41)
..3	0.33 (0.10 to 0.56)
.. → displaced	0.42 (0.09 to 0.75)
.2	0.50 (0.32 to 0.69)
..2	0.36 (0.13 to 0.58)
.. → displaced	0.30 (0.02 to 0.57)
.. → long minor	0.59 (0.23 to 0.96)
..3	0.27 (–0.04 to 0.58)
.3	0.61 (0.40 to 0.82)
..1	0.66 (0.23 to 1.00)
..2	0.00 (0.00 to 0.00)
..3	0.44 (0.18 to 0.70)
.. → diaphysis	0.14 (–0.13 to 0.41)
.. → trochanter major	0.14 (–0.12 to 0.41)
B	0.96 (0.93 to 0.99)
.1	0.82 (0.73 to 0.92)
..1	0.89 (0.67 to 1.00)
..2	0.49 (0.07 to 0.92)
..3	0.80 (0.69 to 0.91)
.2	0.73 (0.61 to 0.84)
..1	0.32 (0.08 to 0.56)
.. → displaced	0.21 (–0.04 to 0.46)
..2	0.23 (0.00 to 0.46)
.. → displaced	0.19 (–0.06 to 0.44)
..3	0.63 (0.43 to 0.83)
.. → displaced	0.62 (0.38 to 0.86)
.3	0.36 (–0.01 to 0.72)

### Network performance

**Figure 3:**
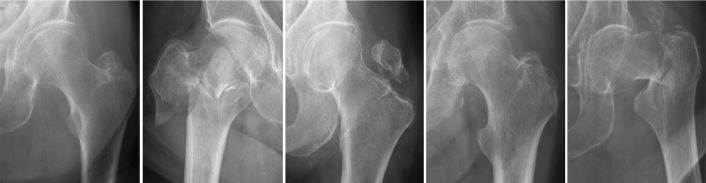
A series of radiographic images classified under group 31A. Despite the advanced capabilities of our AI model, these images highlight instances where the system did not accurately classify the fractures. Each case in this collection was incorrectly categorized by the network, underscoring the challenges and complexities inherent in fracture identification. The inclusion of these examples serves to illustrate the current boundaries of AI accuracy and the need for ongoing model training and improvement.

**Figure 4:**
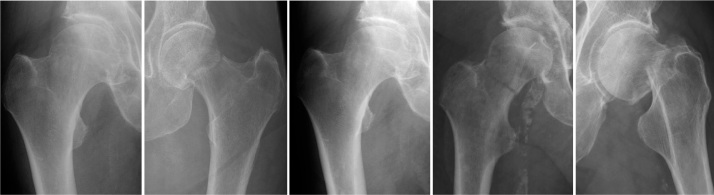
A set of radiographic images from group 31B. These particular cases represent scenarios where the AI model’s classification did not align with the established gold standard. The images are provided to demonstrate the limitations encountered by the network, reflecting the sophisticated nature of fracture classification. Reviewing these misclassified cases offers valuable insights into the model’s performance and informs future directions for algorithmic enhancements.

In the test set, the evaluation threshold for fracture categories was set to a minimum of 4 cases. In general, the network performed well, with 29 out of 39 categories achieving an AUC ≥ 0.95 (Table 3). The sensitivity and specificity for detecting hip fractures were 96% and 94%, respectively. The AUC was 0.99, CI 0.98–0.99. The network performed well across different AO-OTA classes and fracture descriptors, with high sensitivity and specificity. This is particularly important in the merged groups, where treatment decisions are influenced by the accurate identification of fracture types. In the process of developing our AI model, we found that employing a detailed categorization scheme wherein a multitude of specific fracture types were individually identified, contrary to expectations, optimized the model’s learning process. This counterintuitive approach is predicated on the principle that detailed, contextual learning targets enhance the network’s ability to prioritize and learn from a diversified feature set. Thus, while clinically related categories were combined for interpretive clarity, the AI was trained with a wide array of distinct categories to sharpen its predictive accuracy. In the merged A12 or B3 group, the sensitivity and specificity were 91% and 95%, respectively, while the AUC was 97%, CI 0.95–0.98. In the merged A22, A23, or A33 group, the sensitivity was 88%, the specificity was 92%, and the AUC was 0.94%, CI 0.91–0.96. The network also performed well in identifying fractures within custom modified subcategories. For the “displaced,” “trochanter major,” and “long minor” fracture subcategories, the combined sensitivity and specificity were high, with values generally exceeding 90%. The AUC for these categories ranged from 0.93 to 0.97, with 95% confidence intervals typically within the range of 0.90 to 0.99. These results indicate that the network performed well in identifying the fracture types within these crucial merged groups and custom modified subgroups, which is essential for ensuring appropriate treatment and patient care. Examples of misclassified cases are illustrated in Figures 3 and 4 in the Appendix.

**Table 3:** Network’s results for hip fractures (N = 343)

Site^a^	n	Sensitivity (%)	Specificity (%)	Youden’s J	AUC (CI)
Hip	156	96	94	0.89	0.99 (0.98–0.99)
A (Intertrochanteric)	72	92	93	0.85	0.98 (0.96–0.99)
.1	34	91	83	0.74	0.93 (0.89–0.96)
..1	9	100	85	0.85	0.95 (0.91–0.99)
..2	11	100	83	0.83	0.95 (0.91–0.99)
..3	14	100	88	0.88	0.96 (0.93–0.98)
…displaced	9	100	87	0.87	0.94 (0.90–0.97)
…troch. major	8	100	86	0.86	0.93 (0.88–0.98)
.2	23	100	90	0.90	0.97 (0.95–0.99)
..2	16	100	90	0.90	0.96 (0.93–0.98)
…displaced	9	100	88	0.88	0.94 (0.91–0.98)
…long minor	5	100	86	0.86	0.94 (0.89–0.99)
..3	7	100	90	0.90	0.95 (0.92–0.99)
.3	15	93	83	0.76	0.94 (0.88–0.99)
..3	11	91	85	0.76	0.91 (0.84–0.98)
…displaced	4	100	90	0.90	0.96 (0.92–1)
…troch. major	8	100	83	0.83	0.92 (0.88–0.97)
B (Femoral neck)	84	99	97		0.96 (0.99–1)
.1	40	98	87	0.85	0.94 (0.91–0.96)
..1	5	100	81	0.81	0.93 (0.86–0.99)
..3	32	97	86	0.83	0.94 (0.92–0.97)
.2	40	98	85	0.82	0.93 (0.91–0.96)
..1	10	100	75	0.75	0.86 (0.80–0.91)
…displaced	8	100	81	0.81	0.89 (0.84–0.94)
..2	18	100	78	0.78	0.91 (0.88–0.95)
…displaced	15	93	85	0.78	0.92 (0.88–0.96)
…3	12	100	80	0.80	0.90 (0.86–0.95)
…displaced	10	100	87	0.87	0.93 (0.89–0.96)
.3	4	100	87	0.87	0.95 (0.89–1)
Merged groups					
A12 or B3	15	93	86	0.79	0.94 (0.90–0.98)
A22, A23, or A33	34	97	91	0.88	0.95 (0.91–0.98)
B displaced	65	98	96	0.95	0.99 (0.99–1)

a See Table 1

n = the number of fractures observed by the reviewers.

Other fractures were also detected, such as femur diaphyseal fractures and pelvic fractures (Table 4). The sensitivity ranged from 75% to 100%, and the specificity ranged from 78% to 94%. The AUC values ranged from 0.87 to 0.95, indicating good performance in detecting these fractures.

**Table 4:** Network performance on femoral shaft and pelvic fractures (N = 343)

Site^a^	n	Sensitivity (%)	Specificity (%)	Youden’s J	AUC (CI)
Diaphyseal femur	14	86	94	0.80	0.95 (0.90–0.99)
A	9	89	85	0.74	0.94 (0.87–1)
.1	8	75	94	0.69	0.88 (0.75–1)
B	4	100	78	0.78	0.92 (0.82–1)
Pelvis					
A	18	83	80	0.63	0.86 (0.77–0.96)
.2	18	83	81	0.64	0.87 (0.77–0.96)
..2	17	82	85	0.67	0.86 (0.76–0.96)

a See Table 1

n = the number of fractures observed by the reviewers.

## Discussion

We aimed to train and evaluate an AI-based CNN to identify and classify hip fractures according to the AO-OTA 2018 classification system. The network demonstrated high sensitivity and specificity in detecting fractures across different anatomical locations and AO-OTA classes. It showed promising results for identifying fracture types within crucial merged groups, which is of great importance for determining the appropriate treatment approach and improving patient outcomes. Although the study primarily focused on hip fractures, resulting in fewer cases of femoral shaft and pelvis fractures, the network still showed reasonable performance in these areas. The results tended to vary more within an AO-OTA category and between different bone segments than they did between different categories. This variation may be attributed to factors such as insufficient training cases, with categories containing fewer cases often underperforming compared with more common categories.

Previous studies regarding DL in trauma orthopedics have reported high AUC values, which may seem too ideal as not all fractures correspond perfectly to a single class, and borderline cases leave room for different interpretations and classifications [11,21,22]. Accuracy was a major comparison measurement in these studies, but it is directly dependent on the distribution of the test dataset. Some studies removed or excluded images that were difficult to classify, which may introduce selection bias and inflate performance metrics [12,13].

Different users may have varying classification interpretations, and radiographic images contain more information than a single classification system can cover. Our study used a dataset of 6,361 images, which is larger than most studies in this field [11,14]. However, most published studies, including this study, were conducted in a single institution, potentially limiting generalizability.

Establishing a ground truth is a main challenge in this field, and several methods can be used, such as involving experts in orthopedics and musculoskeletal radiology or utilizing MRI or CT scans. Nevertheless, even with these resources, different image interpretations can still pose challenges [11,21]. We fully acknowledge that CT imaging could serve as a superior gold standard for fracture classification. However, due to practical considerations such as the extensive time required for CT review, the absence of routine CT usage in Swedish hip fracture treatment protocols, budgetary constraints, and the vast number of exams, we did not include CT in our classification process. Despite these challenges, the neural network demonstrated satisfactory performance, suggesting an intrinsic capability to discern and learn from the data provided.

The use of AI may have some negative consequences, such as automation bias, which refers to overreliance on clinical decision support systems [10]. The potential downside is that clinicians may become too dependent on AI-based systems, affecting their judgment or learning ability. The “AI black box” issue arises as we do not know which features were used by the machine to produce the prediction [7,8,23], complicating the incorporation of AI into everyday clinical settings, and then there is the matter of accountability using complex AI-based systems.

### Strengths and limitations

A key strength of this study was the inclusion of images with distractions, such as implants, older fractures, and pathologies like tumors, reflecting a more realistic clinical setting. However, this approach also proved disadvantageous, as having more outcome categories results in fewer cases in each category.

We did not conduct a direct comparison between network performance and clinicians’ performance. The study’s performance may have been influenced by factors such as using a less controlled environment for training and testing compared with other previous studies [22]. We believe striving for perfect network accuracy may not translate well into real-world settings and could introduce selection bias.

One limitation is the lack of implementation of other imaging modalities such as MRI or CT, which could have aided the research team in interpreting the images. The limited timespan and the lack of prospectively collected data, where perioperative classification of the fractures is considered the gold standard, are also limitations. Furthermore, the training set was evaluated by a medical student who may have lacked expertise, and the absence of double-checking the training set likely lowered the network’s performance.

Another limitation was the lack of patient medical history, concurrent medications, radiographic referrals, and symptoms like pain location and physical examination, which could help radiologists and other clinicians. However, this also could yield a more unbiased evaluation because no other factors, other than the images and radiologists’ reports, could influence the classification results.

Finally, external validity is a limitation factor, as all images on which the network trained were collected over a decade from only 1 hospital in Stockholm, Sweden. This could pose limitations, as image interpretation and fracture classification could differ in other hospitals, cities, and countries using different radiographic equipment and image file formats.

### Conclusions

The aim of this study was to develop and validate a convolutional neural network (CNN) for the automated classification of hip fractures based on the 2018 AO-OTA classification system. The results demonstrated that the CNN performed well, with AUCs ranging from 0.76 to 0.99 across different fracture categories, indicating high accuracy and reliability in classification. These findings suggest that, with further training and modification, the CNN can be utilized effectively in clinical settings to aid in the interpretation of plain hip radiographs.

### Clinical applications and future studies

The AI-based CAD system developed in this study shows promise for improving diagnostic accuracy and efficiency in clinical settings, especially where radiologist availability is limited. Future studies should focus on validating the AI model in diverse clinical environments and evaluating its impact on clinician performance. Integrating AI tools with clinical expertise could enhance diagnostic workflows and support more informed treatment planning.

## Notes

Conceptualization: MG, EA. Methodology: MG, EA, MM. Software: MG, ASR. Validation: MeM, EA, MG, OL. Formal analysis: MeM, EA, MG, MM. Investigation: MeM, ET, OL, MG. Resources: MG, MM. Data curation: MG, MM. Writing original draft: EA, MeM. Visualization: EA, MM. Supervision: MG, MM. Review and editing: all the authors.

Handling co-editors: Jeppe Vejlgaard Rasmussena and Robin Christensen

*Acta* thanks Olivier Quinten Groot and Henrik Palm for help with peer review of this manuscript.
